# Structural, Optical, and Arsenic Removal Properties of Sol–Gel Synthesized Fe-Doped TiO_2_ Nanoparticles

**DOI:** 10.3390/nano12193402

**Published:** 2022-09-28

**Authors:** Francisco Gamarra, Jesús Medina, Wilson Lanchipa, Rocío Tamayo, Elisban Sacari

**Affiliations:** 1 Laboratorio de Nanotecnología, Facultad de Ingeniería, Universidad Nacional Jorge Basadre Grohmann, Av. Miraflores s/n, Tacna 23003, Perú; 2 Departamento de Ingeniería de Materiales, Facultad de Ingeniería de Procesos, Universidad Nacional de San Agustín, Arequipa 04001, Perú; 3 Laboratorio de Microscopia Electrónica de Transmisión, Centro de Microscopia Electrónica, Facultad de Ingeniería de Procesos, Universidad Nacional de San Agustín, Arequipa 04001, Perú

**Keywords:** arsenic, adsorption, titanium, iron, doping

## Abstract

Pure and Fe-doped TiO_2_ nanoparticles were synthesized by the sol–gel method. The samples were characterized by X-ray diffraction, Raman spectroscopy, BET, UV-vis diffuse reflectance spectroscopy, and scanning electron microscopy. The results show a dependence between the crystallite size and the amount of dopant, which decreases from 13.02 to 12.81 nm. The same behavior was observed in the optical properties, where the band gap decreased from 3.2 to 2.86 eV. The arsenic (V) adsorption was tested in aqueous solution containing 5 mg/L of arsenic and 0.5 g/L of adsorbent at pH 7 and in dark conditions. The results indicate that the TiO_2_-B sample shows a higher arsenic removal, reaching 88% arsenic removal from the water at pH 7. Thus, it is also shown that the best performance occurs at pH 5, where it reaches an arsenic removal of 94%. Ion competition studies show that arsenic removal capacity is slightly affected by chloride, carbonate, nitrate, and sulfate ions. According to the results, the synthesized samples are a promising material for treating arsenic-contaminated water.

## 1. Introduction

Arsenic is a naturally occurring element that is found in the air, soil, and water [1]. The presence of dissolved arsenic in drinking water and groundwater has been reported in a number of countries, but the problem is particularly severe in Bangladesh, India, and other Southeast Asian countries [2,3]. The presence of arsenic has also been reported in Latin America, in countries such as Argentina [4], Bolivia [5], Chile [6], Colombia [7], Brazil [8], Peru [9], and others [10]. The presence of arsenic in Peru is linked to mining activities [9] and to the presence of numerous volcanic systems and geothermal systems, which are characterized by high concentrations of arsenic and other geothermal elements such as boron [11]. Particularly in Tacna, located in southern Peru, arsenic concentrations of up to 0.5 mg/L have been reported in drinking water, which has been attributed to a natural contamination due to the lixiviation of arsenic from volcanic rocks in the course from the water catchment points to the reservoirs [12]; this concentration is higher than recommended by the World Health Organization (10 μg/L) [13,14].

Arsenic is a carcinogen, and chronic exposure to contaminated drinking water significantly increases the risk of developing skin [15], lung [16], bladder [17], and kidney [18] cancers. Due to arsenic’s high toxicity, numerous technologies have been developed to remove arsenic from contaminated water, including flocculation [19], ion exchange [20], membrane filtration [21], reverse osmosis [22], adsorption [23], and other techniques [24].

Each of the aforementioned processes has distinct advantages and disadvantages, making it difficult to choose the most appropriate one. The disadvantages of traditional methods include their high cost, high sludge production, membrane fouling (nanofiltration), and the need for continuous monitoring of ion concentrations (ion exchange) [25]. When the disadvantages of the various processes are considered, adsorption is one of the most widely used ways of removing arsenic from aqueous solutions and is now considered a cost-effective and efficient form of water treatment [23].

Numerous studies have investigated the removal of arsenic using iron oxide [26], copper oxide [27], titanium dioxide [28], zeolite [29], perovskites [30], chitosan [31], and other materials as adsorbents [32]. Among these adsorbents, titanium dioxide has been widely used in arsenic removal due to its advantageous properties, including low cost, nontoxicity, and environmental friendliness, as well as optimal optical and electronic properties [28]. In order to improve the performance of titanium dioxide, different types of modifications have been studied through doping [33], compound development [34,35,36], and structural and morphological modification [37], but these have been applied mainly in heterogeneous photocatalysis for organic pollutants’ photodegradation [38].

Doping enables the modification of the crystal structure, optical properties, and chemical composition, but according to the bibliographic review, there is scarce information about how iron doping impacts TiO_2_ arsenic removal capacity from water [39,40].

Due to the similar radius of Fe^3+^ (0.64 Å) and Ti^4+^ (0.68 Å), iron metal ions were regarded as a suitable dopant in this work [41]. As a result, it integrates easily into the TiO_2_ crystal lattice. One of the advantages of incorporating Fe into the TiO_2_ lattice is that it improves the arsenic removal capacity of the TiO_2_ [40,42].

The objectives of this research were to synthetize Fe (III)-doped TiO_2_ nanoparticles with different concentrations using a sol–gel method, characterize them, and determine the effect of iron doping on structural, vibrational, optical, morphological, and arsenic removal properties of pure and Fe-doped TiO_2_. The properties of prepared materials were characterized using X-ray diffraction (XRD), Raman spectroscopy, UV-visible spectroscopy, FTIR spectroscopy, scanning electron microscopy, transmission electron microscopy, as well as nitrogen physisorption studies at 77 K. We conducted batch tests to determine the arsenic sorption properties of synthesized materials.

## 2. Materials and Methods

### 2.1. Synthesis of TiO_2_ Nanoparticles

The pure TiO_2_ nanoparticles were prepared by the sol–gel method as follows. First, 4.06 mol (28 mL) of glacial acetic acid (Merck, Darmstadt, Germany,) and 5.6 mol (52 mL) of 2-propanol (Merck) were mixed and stirred for 10 min, then 0.67 mol (24 mL) of titanium (IV) isopropoxide (97%, Sigma Aldrich, Steinheim, Germany) was dissolved and stirred for 30 min. After that, 7.8 mol (17 mL) of water was added to form the gel. The gel was dried at 40 °C overnight; the obtained powder was grinded in an agate mortar for 30 min. The fine powder was calcined at 400 °C for 2 h, thereby obtaining the pure TiO_2_ nanoparticles.

### 2.2. Synthesis of Iron-Doped TiO_2_

Iron-doped TiO_2_ nanoparticles were synthetized mixing 4.06 mol (28 mL) of glacial acetic acid (Merck) and 5.6 mol (52 mL) of 2-propanol (Merck) for 10 min; afterward, that solution was divided in two, into 60 and 20 mL, respectively. In the first one, 0.67 mol (24 mL) of titanium (IV) isopropoxide was added and stirred for 30 min; in the second solution, different amounts of iron (III) chloride hexahydrate (100, 200, and 300 mg) were added and stirred for 30 min. Each solution was added individually to the first solution dropwise and stirred additionally for 30 min. Finally, 7.8 mol (17 mL) of deionized water were added to each solution, forming a gel. The obtained gels were dried at 40 °C overnight, then grinded in an ágata mortar for 30 min and calcined at 400 °C for 2 h. Each sample was labeled as TiO_2_-A, TiO_2_-B, or TiO_2_-C, according to the iron amount (100, 200, and 300 mg, respectively).

### 2.3. Characterization

The thermal behavior at a constant heating rate of 20 °C/min in N_2_ atmosphere of as-prepared dried samples were analyzed using a simultaneous thermal analyzer, Discovery SDT 650 (TA Instruments, New Castle, DE, USA). The structural characterization of pure and doped TiO_2_ nanostructures was performed on a PANalitycal X-ray diffractometer model AERIS Research (Malvern Panalytical Ltd.a., Almedo, The Netherlands) using Ni-filtered CuK_α_ radiation (1.5406Å) operated at 40 kV and 15 mA. The diffraction patterns were recorded from 20° to 80° with steps of 0.022° and 20 s per step. The crystallite size and structural parameters were determined by Rietveld refinement using X’Pert HighScore Plus Software V. 4.9. UV-visible diffuse reflectance spectra (DRS) of the powder samples were recorded from 200 nm to 700 nm using Thermo Scientific spectrometer model Evolution 220 (Thermo Scientific Co., Ltd., Waltham, DE, USA), equipped with an integration sphere of 10 cm diameter (ISA-220) and using white Spectralon disc as blank. The specific surface area (SSA_BET_) of the nanostructures was measured by nitrogen adsorption at 77 K using a Micromeritics surface analyzer model Gemini VII 2390t (Micromeritics Instrument Co., Norcross, GA, USA) after degassing the sample for 1 h at 150 °C under helium fluctuation. Attenuated total reflectance (ATR) Fourier-transform infrared (FTIR) spectra were taken using a Perkin Elmer Frontier Model Spectrometer (PerkinElmer Inc., Wellesley, MA, USA). Sample morphology was observed by field emission scanning electron microscope (FE-SEM) model Quattro S (Thermo Scientific Co., Eindhoven, The Netherlands), operated at 30 kV and high vacuum, and transmission electron microscope model Talos F200i (Thermo Scientific Co., Eindhoven, The Netherlands) operated at 200 kV. The arsenic concentration in all the samples was measured using a Shimadzu Atomic Absorption Spectrometer model AA-6300 equipped with graphite furnace atomizer (GFA-EX7i) (Shimadzu Scientific Instruments, Inc., Kyoto, Japan), with a hollow cathode lamp (293.7 nm) operated at 25 mA and using a Deuterium lamp as background correction.

### 2.4. Batch Adsorption Experiments

Stock arsenic (V) solution (5 ppm) was prepared by dissolving 0.0208 g of di-sodium arsenate (Na_2_HAsO_4_·7H_2_O) (Merck) in 1 L of deionized water.

Batch adsorption experiments were carried out to estimate the arsenic adsorption capacity of pure and doped TiO_2_ nanoparticles. Typically, 50 mg/L of adsorbent was mixed in a 100 mL of 5 ppm arsenic-containing water in a glass vessel and agitated at 200 rpm for 2 h at 25 °C. The adsorption experiments for arsenic removal were conducted under dark conditions. The samples were taken at different time intervals, centrifuged at 4000 rpm for 5 min, and analyzed by graphite furnace atomic absorption spectroscopy.

### 2.5. pH Influence on the Arsenic Removal

To determine the influence of the pH on the arsenic removal efficiency, some solutions with 5 ppm of arsenic concentration at different pH were prepared. The pH was adjusted by the addition of dilute hydrochloric acid or sodium hydroxide solutions. Samples were collected after 2 h of the adsorption process in dark conditions and centrifuged at 4000 rpm for 5 min. The supernatant was analyzed by atomic absorption spectroscopy equipped with a graphite furnace.

### 2.6. Effect of Competitive Ions on the Arsenic Removal

Dissolved anions such as chlorides ($Cl^{-}$), carbonates ($CO_{3}^{2-}$), nitrates ($NO_{3}^{-}$), and sulfates ($SO_{4}^{2-}$) have been reported to reduce the arsenic removal capacity of different adsorbents.

To study the influence of the competition ions, solutions containing various ions were prepared using NaCl, Na_2_CO_3_, NaNO_3_, and Na_2_SO_4_ salts at different concentrations (10, 25, and 50 ppm)

The experiments were carried out at 5 ppm arsenic concentration and 50 mg/L of adsorbent; the pH was adjusted to the desired value (~7.0) using NaOH solution.

Samples were collected at known intervals and centrifuged at 4000 rpm for 5 min. The supernatant was analyzed by atomic absorption spectroscopy equipped with a graphite furnace.

## 3. Results and Discussion

### 3.1. Thermal Analysis

**Figure 1:**
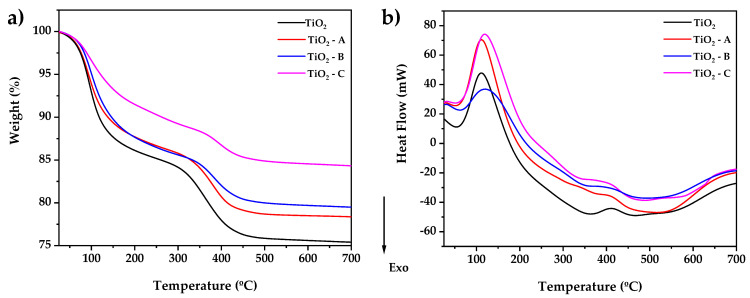
(**a**) Thermogravimetric analysis and (**b**) scanning differential calorimetry of dried pure and doped TiO_2_ samples.

Thermogravimetric analysis of TiO_2_-, TiO_2_-A-, TiO_2_-B-, and TiO_2_-C-reserved samples after gel-drying at 40 °C overnight is shown in Figure 1a. The TGA curve exhibits four mass losses associated with endothermic and exothermic events that occur in the DSC curve (Figure 1b). The first endothermic event that occurs at 55–160 °C corresponds to the elimination of the remaining 2-propanol and adsorbed water present in the samples and adsorbed water, with a weight loss of 16, 12, 12, and 8%, respectively. There is then an exothermic drop of 160–350 °C that is related to the volatilization and combustion of the hydration water and organic species such as Pr^i^OH and CH_3_COOH, with a weight loss of 19, 16, 15, and 12%, respectively. The endothermic peak in the DSC curve between 350 and 490 °C roughly corresponds to the crystallization of the amorphous residue to the anatase TiO_2_ phase. This process caused a weight loss of 25, 22, 20, and 15%, respectively, followed by a slight endothermic slope in the range of 490–550 °C, indicating crystalline growth, and finally followed by an endothermic shoulder between 550–700 °C, indicating the anatase–rutile phase transition, with a total weight loss of 25, 22, 20, and 15% at the end of the process. The results show that the weight loss decreases as the amount of doping increases. In the same way, the temperature and the heat flow necessary for the crystallization of titanium in the amorphous phase to the anatase phase are reduced.

### 3.2. X-ray Diffraction

**Figure 2:**
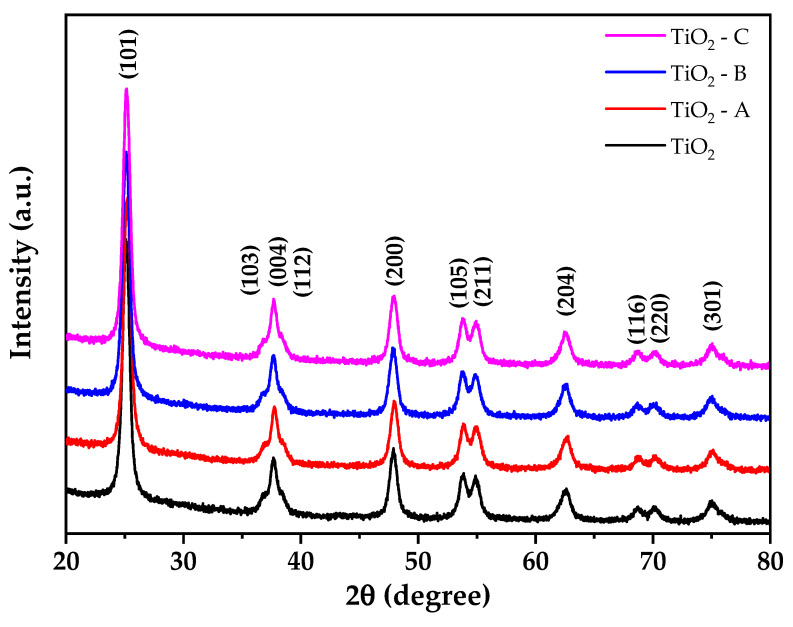
X-ray diffraction patterns of pure and doped TiO_2_.

Figure 2 displays The X-ray diffraction (XRD) result of pure and doped TiO_2_. The material exhibits a tetragonal structure, and the patterns are well-matched with JCPDS 01-071-1168, corresponding to the anatase phase. The crystallite size and lattice parameters of the prepared materials were calculated by Rietveld refinement using PANAlytical Higscore Plus scientific software V4.9. The diffraction data backgrounds were modeled using the Chebishev method, and the Pseudo Voight profile function was used to generate the peak profile. The tetragonal anatase phase of TiO_2_ was used to index all the peaks. Table 1 summarizes the outcomes of the refinements. Additionally, it demonstrates that pure and doped TiO_2_ samples present an anatase phase (100%) tetragonal structure, with no contribution from rutile or brookite phases.

These diffractograms reveal no additional peaks, indicating that the anatase phase is not distorted during doping. However, increasing the amount of iron doping results in small changes in the intensity and width of the diffraction peaks. These could be a result of crystallite contraction (Table 1) due to the doping of Fe^3+^ by substitution in the crystal lattice of TiO_2_ [43].

The absence of additional titania phases at greater concentrations of dopant indicates that phase transitions are inhibited [44]. The results also show that the samples exhibit crystallite sizes between 12.91 and 13.02 nm, where the crystallite size of pure TiO2 is larger than the doped samples due to the replacement of Ti^4+^ ions for Fe^3+^ ions, which results in the deformation of TiO_6_ octahedra [45]. An N_2_ adsorption test at liquid nitrogen temperature was conducted to analyze the surface area of pure TiO_2_ and Fe-TiO_2_ samples (Table 1). The specific surface area (SSA_BET_) of TiO_2_ was decreased after iron doping, especially for samples TiO_2_-A and TiO_2_-C. However, TiO_2_-B showed a higher specific surface area (102.25 ± 0.98 m^2^/g), which can be beneficial for arsenic removal tests from water because of the greater contact area.

### 3.3. Raman Spectroscopy

**Figure 3:**
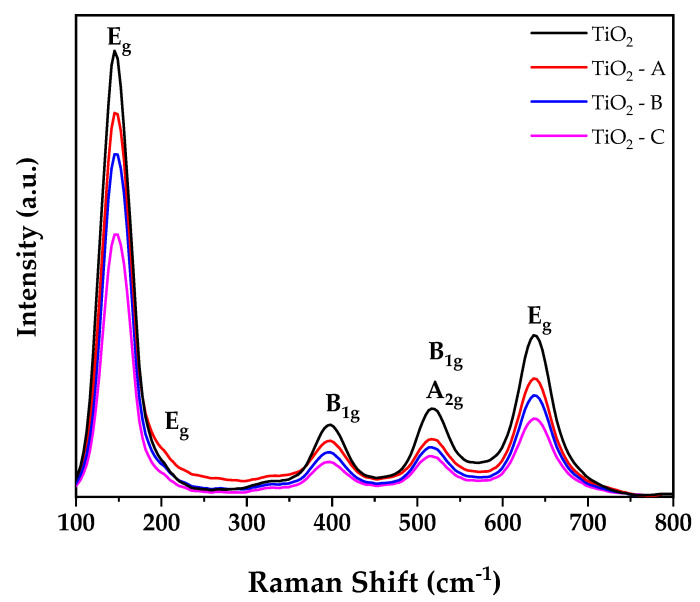
Raman spectra of spectra of pure and doped TiO_2_.

Figure 3 illustrates the Raman spectra of pure and doped TiO_2_ samples. All samples exhibit Raman bands at 145, 197, 398, 516, and 636 cm^−1^ that are quite similar to Raman bands of TiO_2_ anatase phase single crystal (144, 196, 394, 516, and 638 cm^−1^) [31,46]. The first and second bands correspond to the E_g_ vibration mode, whereas the next three bands correspond to the B_1g_, A_2g_, and E_g_ vibration modes of anatase phase TiO_2_. No additional bands were detected for iron oxides, indicating the existence of a dopant cation in the TiO_2_ crystal lattice’s substitutional locations. The observed results corroborate the XRD results. Additionally, it is found that when the iron doping concentration in the TiO_2_ crystal lattice increases, the intensities of peaks at 145 cm^−1^ decrease with an increase in a dopant in full width half maximum (FWHM). This result can be attributed to a decrease in the crystalline size of TiO_2_ structure and an increase in oxygen deficit [47].

### 3.4. UV-Visible Spectroscopy

**Figure 4:**
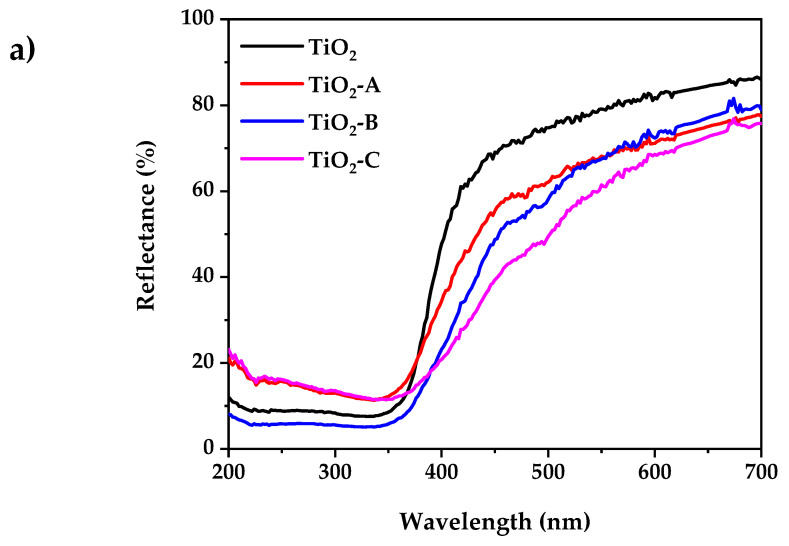

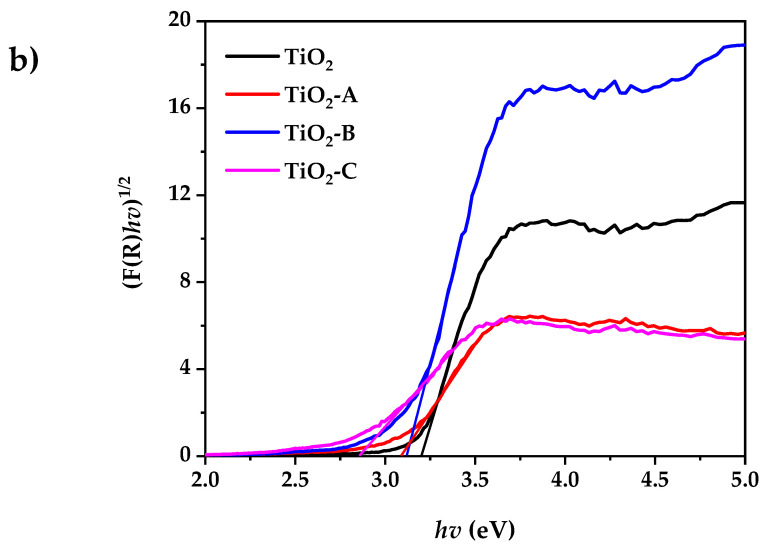
(**a**) UV-visible diffuse reflectance spectrum, (**b**) Kubelka–Munk plot for bandgap calculation.

Figure 4a shows the UV-visible diffuse reflectance spectra of pure and Fe-doped TiO_2_. According to the experimental results, the pure and doped TiO_2_ powder exhibits low reflectance at wavelengths less than 400 nm. The reflectance spectra of all the samples in the visible range decreases with the increased dopant concentration. This is accompanied by a change in color from white (pure TiO_2_) to pale yellow (Fe-doped TiO_2_). This indicates that the band gap has narrowed. The indirect bandgap (E_g_) of pure and doped TiO_2_ samples were determined using the Kubelka–Munk equation, as shown in Figure 4b:

$$F\left(R\right)=\frac{K}{S}=\frac{{\left(1-R\right)}^{2}}{2R}$$

where *F(R)* is the Kubelka–Munk function, *K* is absorption coefficient of radiation, *S* is the scattering factor, and *R* is the ratio of the intensities of radiation reflected in a diffuse [48]. The bandgap of pure and doped TiO_2_ samples are summarized in Table 1.

The results show that the increase in dopant concentration decreases the TiO_2_ band gap energy from 3.2 to 2.86 eV. This can be attributed to the creation of a dopant level closer to the valance band, resulting in the excitation of Fe^3+^ 3d electrons into the TiO_2_ conduction band (charge transfer transition) [49].

### 3.5. Fourier-Transform Infrared Spectroscopy (FTIR)

**Figure 5:**
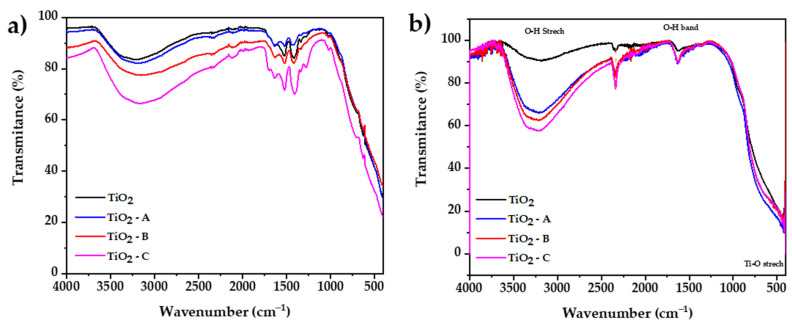
FTIR spectrums of (**a**) prepared dried gel and (**b**) calcined samples.

The FTIR spectroscopy method was used to investigate the vibrational bands on synthetized samples. Figure 5a shows that between 3700 and 2300 cm^−1^, a broad band may be attributed to the stretching vibration mode of the single bond OH group, which exhibits coordination vacancies when exposed to water. The results also display that the increase in dopant reduces the transmittance, which would be attributed to the presence of additional molecular water present in the iron (III) chloride hexahydrate. A peak located at 1700 cm^−1^ is related to the stretching vibration of the hydrogen-bonded C = O group of acetic acid. Five additional peaks at the 1670 and 1100 cm^−1^ range were observed, which corresponds to vibration modes of the COO group [50]; the increase in the intensity of this peaks is attributed to oxxo groups formation, which increases with the amount of dopant.

Figure 5b shows the FTIR spectrum of calcined samples, respectively. The results display between 3600 and 2400 cm^−1^, and a broad band may be attributed to the stretching vibration mode of the single bond OH group [51], which exhibits coordination vacancies when exposed to water. Iron doping in titania results in an increase in surface hydroxylation, which is reflected in a greater percent absorbance in the infrared spectrum. At 2340 cm^−1^, a small peak is observed that could be attributed to stretching vibration of carboxyl groups originated from residual organic species of used precursors such as titanium isopropoxide, 2-propanol, and acetic acid [52]. A bending vibration band is also observed at 1632 cm^−1^, which is a result of the bending vibrations caused by adsorbed water on the surface of TiO_2_ [53]. Clear evidence of characteristic peaks of the Ti–O and O–Ti–O bond stretching vibrations can be seen around 620 and 530 cm^−1^, respectively [54]. All the FTIR spectra are similar because there are no extra peaks from other phases or compounds in the samples that were analyzed. The only difference is the amount of water that the samples can hold, which depends of the dopant content of each sample [55].

### 3.6. Scanning and Transmission Electron Microscopy Analysis

The morphology and elemental analysis of pure and Fe-doped TiO_2_ nanoparticles were evaluated using scanning electron microscopy (SEM), transmission electron microscopy, and energy-dispersive X-ray spectroscopy (EDX).

**Figure 6:**
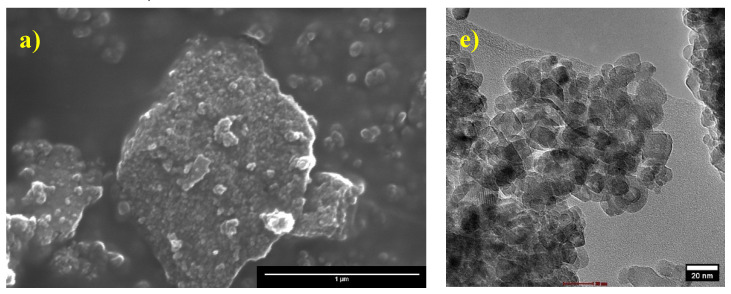

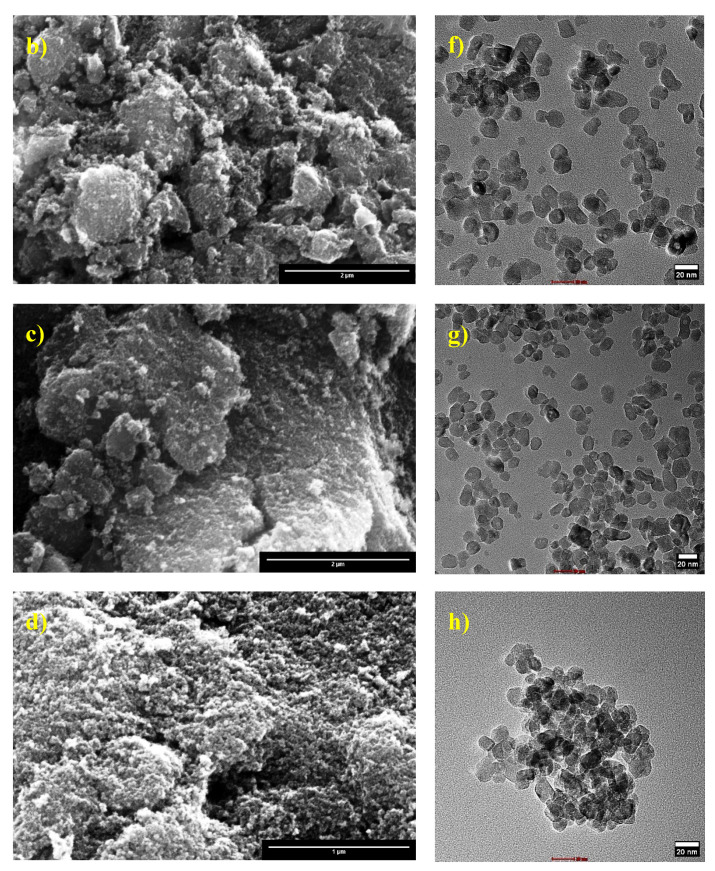
Scanning and transmission electron microscopy of (**a**,**e**) pure TiO_2_, (**b**,**f**) TiO_2_-A, (**c**,**g**) TiO_2_-B, and (**d**,**h**) TiO_2_-C, respectively.

SEM images displayed in Figure 6a–d show pure and Fe-doped TiO_2_ nanoparticles at various dopant concentrations. The images reveal that they are almost agglomerated and irregular in form. As shown in the SEM images, a preliminary estimation of the images indicates that the average particle size is in the nanoscale region. EDX analysis was used to confirm the presence of dopant on TiO_2_. The elemental composition determined by EDX is shown in Table 2. The Fe content of the samples corresponded well with the nominal and observed values.

The results obtained by transmission electron microscopy (TEM) displayed in Figure 6e–h confirm that the particles are irregular and smaller than 100 nm, for which they can be considered nanoparticles. As well as the analysis of multiple images of each of the samples, we have that the average size of the samples is 14.69, 14.56, 13.21, and 13.76 nm, corresponding to the samples of TiO_2_, TiO_2_-A, TiO_2_-B, and TiO_2_-C, respectively (Figure S1).

### 3.7. Arsenic Adsorption Experiment

**Figure 7:**
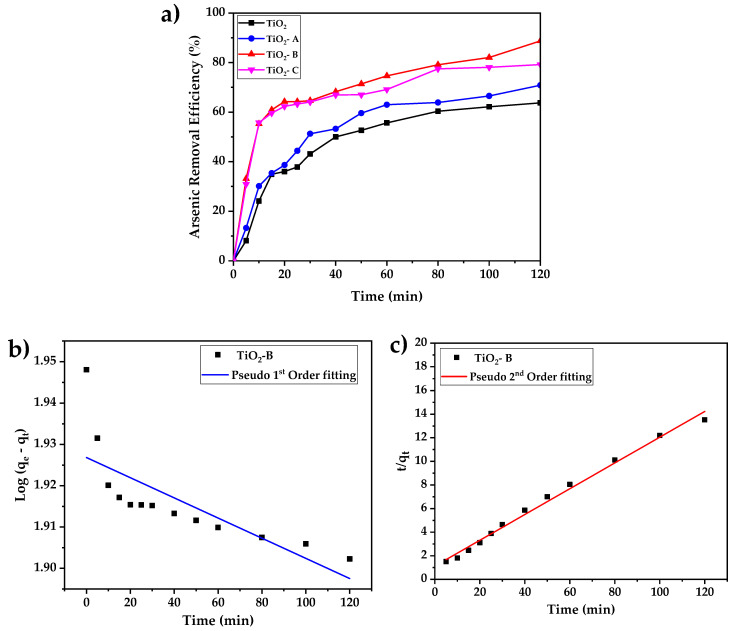
(**a**) Time-dependent adsorption of arsenic by pure and doped TiO_2_ (initial concentration of arsenic 5 mg/L, adsorbent concentration 0.5 g/L and pH = 7), (**b**) linear fit of the kinetic adsorption using pseudo-first-order model, and (**c**) linear fit of the kinetic adsorption using pseudo-second-order model.

The effects of contact time on arsenic adsorption by pure and doped TiO_2_ adsorbents are shown in Figure 7a. The results indicate that increasing the contact time increases arsenic adsorption in all the samples; however, the sample of TiO_2_-B shows improved performance, which could be attributed to its high surface area.

During the first phase of adsorption, which lasts roughly 20 min, the samples TiO_2_, TiO_2_ -A, TiO_2_ -B, and TiO_2_ -C remove approximately 35, 38, 61, and 64% of the arsenic from the solution, respectively. The second phase of adsorption is characterized by a decrease in adsorption efficiency and a nearly constant rate of adsorption. The arsenic removal after 2 h of treatment are 64%, 71%, 79%, and 88%, corresponding to TiO_2_ -A, TiO_2_ -C, and TiO_2_ -B, respectively, with the best performance coming from the TiO_2_-B sample, which can be attributed mainly to its greater surface area.

Arsenic adsorption results were also analyzed using Lagergren’s pseudo-first- (Equation (1)) and pseudo-second- (Equation (2)) order kinetic models, which are commonly used in adsorption experiments [30].

(1) $$\frac{dq_{t}}{dt}=k_{1}\left(q_{e}-q_{t}\right)$$

(2) $$\frac{dq_{t}}{dt}=k_{2}{\left(q_{e}-q_{t}\right)}^{2}$$

where *q_t_* is the removal capacity as a function of time *t*, *q_e_* is the equilibrium adsorption capacity, *k*_1_ and *k*_2_ are pseudo-first- and pseudo-second-order velocity constants, respectively. The linear form of these kinetic models is represented by Equations (3) and (4).

(3) $$log\frac{\left(q_{e}-q_{t}\right)}{q_{e}}=-\frac{k_{1}}{2.303}t$$

(4) $$\frac{t}{q_{t}}=\frac{1}{k_{2}q_{e}^{2}}+\frac{t}{q_{e}}$$

The kinetic parameters obtained are listed in the Table 3. Based on the regression coefficients (*R^2^*), obtained from pseudo-first- and pseudo-second-order fitting, the pseudo-second-order equation proved to be the better-fitting model for the arsenic adsorption of all the samples. It can also be shown that the arsenic adsorption by TiO_2_-B yields the maximum velocity and adsorption capacity, and it is represented in Figure 7b,c.

**Figure 8:**
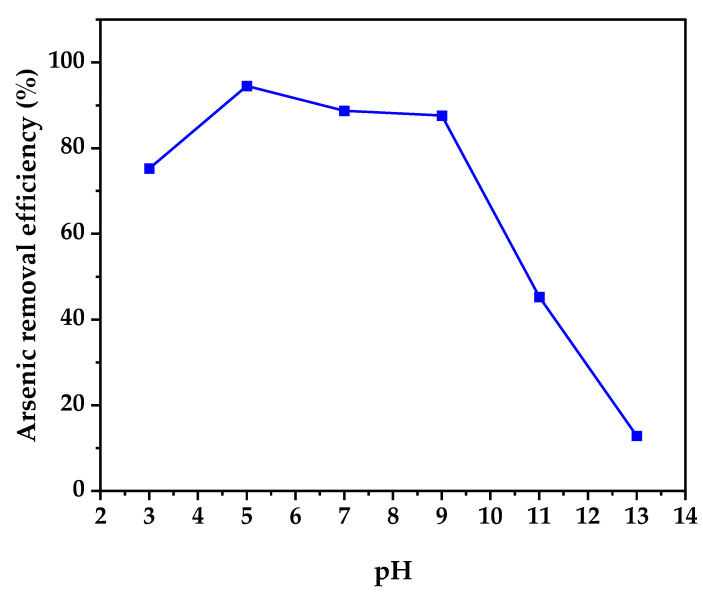
Effect of solution pH on arsenic removal efficiency of TiO_2_-B.

To verify the applicability of the TiO_2_-B sample in various conditions, the sample was evaluated in different pH levels, as shown in Figure 8, and tested under different coexisting ions.

### 3.8. pH Influence on Arsenic Removal

The effect of solution pH and ionic strength on arsenic removal efficiency by TiO_2_-B is seen in Figure 8.

The results show that the TiO_2_-B sample has better performance at pH ranges of 5–9 due to the removal of hydroxyl ions from the coordinating layer on the TiO_2_-B surface, which provides a positive charge on the nanoparticles’ surface and allows them to absorb more arsenic anions from the solution [56]. Outside of that range, the arsenic adsorption decreases rapidly. The arsenic adsorption was around 94% at pH 5, 88% at pH 7, and 87% at pH 9.

### 3.9. Effect of Coexisting Ions

**Figure 9:**
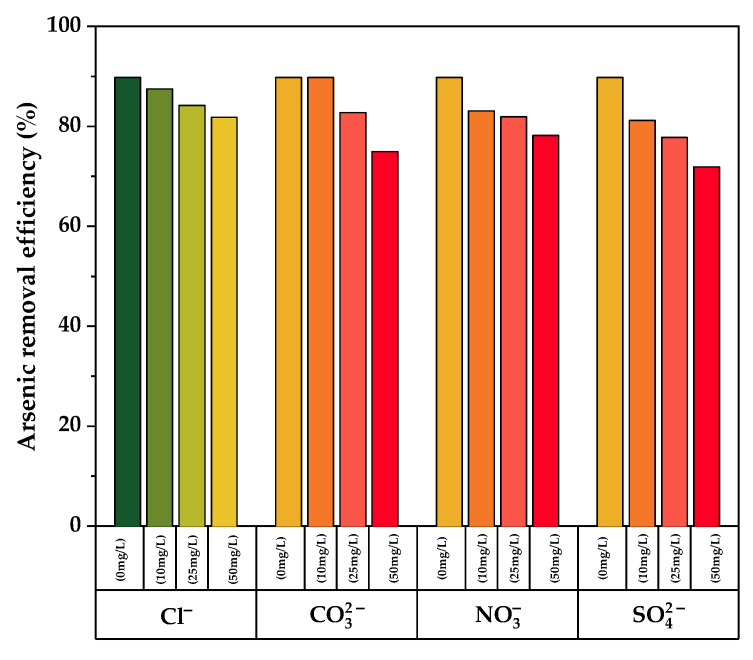
Effect of various competing ions on Arsenic removal by pure and doped TiO_2_-B.

The effect of coexisting ions on arsenic adsorption from aqueous solution was investigated using different concentrations of chloride, carbonate, nitrate, and sulfate ions. As illustrated in Figure 9, an increase in the concentration of coexisting ions results in a decrease in the arsenic removal efficiency for all the measured ions when compared to the blank (no coexisting ions). In the presence of chloride ions, minimal interference was found, but higher interference was observed for sulfate ions, which exhibits a percent adsorption loss of up to 16.7% at its greatest concentration (50 mg/L). As a result, sulfate is the biggest competitor for binding sites on TiO_2_-B with arsenic ions. This is due to the fact that both sulfate and arsenic (V) anions have similar chemistry in aqueous solution at pH 7.0 [57].

## 4. Conclusions

The sol–gel process was used to create pure and iron-doped TiO_2_ nanoparticles. Thermogravimetric analysis demonstrates that weight loss for TiO_2_ and TiO_2_-C is reduced from 22 to 14%, respectively. Differential scanning calorimetry shows that at about 400 °C, a crystallization process associated with the anatase phase formation is observed. According to X-ray diffraction and Raman spectroscopy, a pure anatase phase was formed, with the crystallite size decreasing with iron doping. The addition of Fe also resulted in a reduction in the bandgap from 3.2 to 2.86 eV.

The SEM results show particle agglomeration in all the samples. Transmission electron microscopy images show an irregular shape with a mean particle size of 14.69, 14.56, 13.21, and 13.76 nm for TiO_2_, TiO_2_-A, TiO_2_-B, and TiO_2_-C samples, respectively.

The pseudo-second-order kinetic model was found to best correlate with the data for arsenic adsorption, with a maximal adsorption capacity of 8.67 mg/g, which corresponds to TiO2-B. The influence of several coexisting ions on the arsenic removal capacity of TiO_2_-B reveals that chloride, nitrate, and carbonate ions have minor interference; however, sulfate ions can reduce its capacity by up to 16.7%. These properties demonstrate the high potential of the prepared Fe-doped TiO2 as a nanoadsorbent for efficient arsenic removal from aqueous solution.

## Figures and Tables

**Table 1:** Structural parameters of iron-doped TiO_2_ nanoparticles.

Structural Parameter	Sample			
	TiO_2_	TiO_2_—A	TiO_2_—B	TiO_2_—C
a = b (nm)	3.7843	3.7858	3.7859	3.7862
c (nm)	9.4995	9.5036	9.5051	9.5075
α = β = γ (°)	90	90	90	90
*ρ* (g/cm^3^)	3.9	3.9	3.89	3.89
D (nm)	13.02	12.95	12.81	12.91
R_exp_ (%)	1.7601	1.7665	1.7741	1.7635
R_wp_ (%)	2.5442	2.4332	2.4135	2.3294
R_p_ (%)	2.009	1.9205	1.9184	1.84059
GOF	1.45	1.37	1.36	1.32
E_g_ (eV)	3.2	3.11	3.05	2.86
SSA_BET_ (m^2^/g)	99.78 ± 0.86	98.36 ± 0.74	102.25 ± 0.98	82.94 ± 0.28

**Table 2:** Energy dispersive X-ray spectroscopy (EDX) analysis of all samples.

Element	Sample			
	TiO_2_	TiO_2_—A	TiO_2_—B	TiO_2_—C
Ti (Atom %)	32.19	33.35	32.89	32.22
O (Atom %)	67.81	66.49	66.83	67.36
Fe (Atom %)	0	0.16	0.28	0.42

**Table 3:** Kinetic parameters of pseudo-first-order and pseudo-second-order models calculated from experimental data of pure and Fe-doped TiO_2_.

SAMPLE	Pseudo-First-Order			Pseudo-Second-Order		
	*k*_1_	*q_e_*	*R^2^*	*k_2_*	*q_e_*	*R^2^*
TiO_2_	0.0001755	6.00	0.77896	0.0061776	7.56	0.99596
TiO_2_-A	0.0001636	6.27	0.75018	0.0070052	7.91	0.99607
TiO_2_-B	0.0001224	6.87	0.56323	0.013952	8.67	0.99523
TiO_2_-C	0.0001283	6.53	0.52831	0.0165896	8.20	0.99284

## Supplementary Materials

The following supporting information can be downloaded at: www.mdpi.com/article/10.3390/nano12193402/s1, Figure S1: Particle size distribution of a) TiO_2_, b) TiO_2_-A, c) TiO_2_-B and d) TiO_2_-C.
