# Supplementary material for: Structural, Optical, and Arsenic Removal Properties of Sol–Gel Synthesized Fe-Doped TiO2 Nanoparticles
Source: Nanomaterials (Basel). 2022 Sep 28;12(19):3402. doi: 10.3390/nano12193402 (PMC9565318; doi:10.3390/nano12193402)
Supplement: Supplementary file 1 [file nanomaterials-12-03402-s001.zip › nanomaterials-1907246-supplementary.pdf]

nanomaterials

Supplementary Materials

Structural, Optical, and Arsenic Removal Properties of Sol–Gel Synthesized Fe-Doped TiO_2 Nanoparticles

Francisco Gamarra [1], Jesús Medina [1,*], Wilson Lanchipa [1], Rocío Tamayo [2,3], and Elisban Sacari [1,*]

1 Laboratorio de Nanotecnología, Facultad de Ingeniería, Universidad Nacional Jorge Basadre Grohmann, Av. Miraflores s/n, Tacna 23003, Perú
2 Departamento de Ingeniería de Materiales, Facultad de Ingeniería de Procesos, Universidad Nacional de San Agustín, Arequipa 04001, Perú
3 Laboratorio de Microscopia Electrónica de Transmisión, Centro de Microscopia Electrónica, Facultad de Ingeniería de Procesos, Universidad Nacional de San Agustín, Arequipa 04001, Perú
* Correspondence: jmedinas@unjbg.edu.pe (J.M.); esacaris@unjbg.edu.pe (E.S.); Tel.: +51-959618180 (J.M.); +51-925573684 (E.S.)

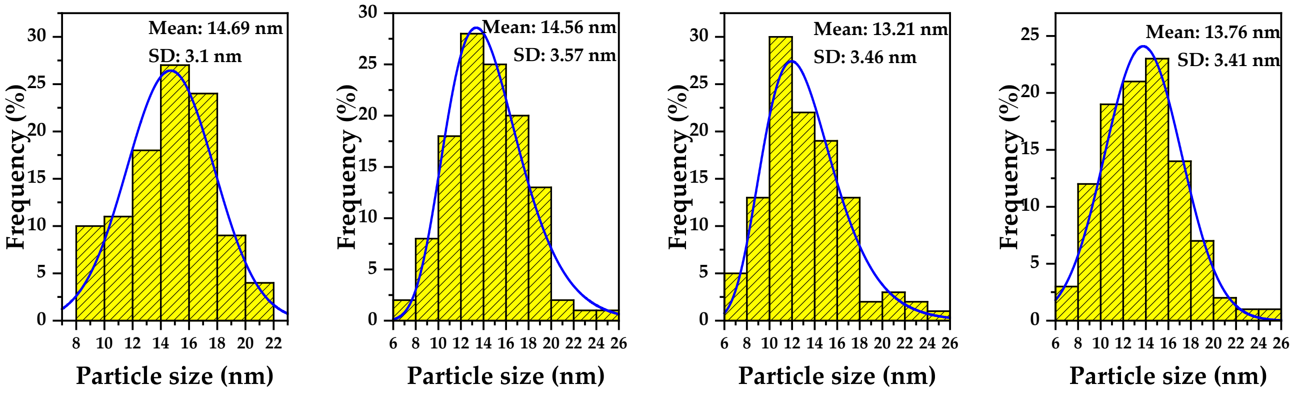

Figure S1. Particle size distribution of (**a**) TiO_2, (**b**) TiO_2-A, (**c**) TiO_2-B and (**d**)TiO_2-C
